# A new species of the ant genus *Recurvidris* Bolton, 1992 (Hymenoptera, Formicidae, Myrmicinae) from Thailand

**DOI:** 10.3897/zookeys.830.31147

**Published:** 2019-03-14

**Authors:** Weeyawat Jaitrong, Yuppayao Tokeeree, Piyaporn Pitaktunsakul

**Affiliations:** 1 Thailand Natural History Museum, National Science Museum, Technopolis, Khlong 5, Khlong Luang, Pathum Thani, 12120 Thailand National Science Museum Pathum Thani Thailand; 2 Program of Environmental Science, Faculty of Science and Technology, Surindra Rajabhat University, Muang, Surin, 32000 Thailand Surindra Rajabhat University Surin Thailand; 3 Department of General Science, Faculty of Science and Technology, Kanchanaburi Rajabhat University, Kanchanaburi, 71190 Thailand Kanchanaburi Rajabhat University Kanchanaburi Thailand

**Keywords:** Formicidae, ants, *
Recurvidris
*, new species, taxonomy, Thailand

## Abstract

*Recurvidris* Bolton, 1992 is a small myrmicine genus of the tribe Crematogastrini. Until now, eleven species are known in this genus from Asia. A new species, *Recurvidrislekakuli***sp. n.**, is here described from Thailand based on the worker caste. The type series of the new species was collected from leaf litter in a dry evergreen forest. A key to the Asian species of *Recurvidris* based on the worker caste is provided.

## Introduction

*Recurvidris* Bolton, 1992 is a small myrmicine genus of the tribe Crematogastrini (Bolton 2003, Ward et al. 2015). Members of the genus are characterized by the propodeal spine curving upwards and forwards from its base; antenna with 11 segments and 3-segmented club; a mandible with 4–5 teeth on the masticatory margin, and the basal margin with or without tooth; and the petiole low and pedunculate (see Bolton 1992). They are distributed in Asia from India and Sri Lanka in the south and west; Japan, China and Taiwan in the north; various countries in Southeast Asia; and eastwards to Sulawesi in Indonesia (Bolton 1992, Xu and Zheng 1995, Zhou 2000, Zettel 2008, Terayama 2009, Jaitrong and Wiwatwitaya 2015, Antweb 2018). Currently, eleven species are recognized in the genus. Among them, Jaitrong and Wiwatwitaya (2015) recorded only three species, *Recurvidrisbrowni* Bolton, 1992; *R.chanapaithooni* Jaitrong & Wiwatwitaya, 2015 and *R.recurvispinosa* (Forel, 1890) from Thailand.

Surveys of ants in Kanchanaburi province, western Thailand under the project “Conservation and economic assessment at Kanchanaburi limestone community forest for sustainable uses”, led to the discovery of a few unidentified *Recurvidris* specimens belonging to the *R.kemneri* species group (sensu Bolton 1992). Having carefully compared them with the type material of closely related species, we concluded that this species is new to science. We here describe and name it *Recurvidrislekakuli* sp. n. based on the worker caste.

## Materials and methods

The material was collected from western Thailand, Kanchanaburi province, Thong Phaphum district, Sahakhon Nikhom village (14.76255556N, 98.80966667E). The area was covered with a dry evergreen forest. The holotype and paratypes of *Recurvidrislekakuli* sp. n. are pin-mounted dry specimens. The type material of the new species was compared with the holotype and paratypes of the most closely related species, *Recurvidrischanapaithooni* Jaitrong & Wiwatwitaya, 2015 (in Natural History Museum of the National Science Museum, Thailand). Most morphological observations were made with a ZEISS Discovery V12 stereoscope.

Multi-focused montage images were produced using NIS-Elements-D from a series of source images taken by a Nikon Digital Sight-Ri1 camera attached to a Nikon AZ100M stereoscope. The holotype and paratypes were measured for the following parts using a micrometer (accurate to 0.01 mm).

The abbreviations used for the measurements and indices are as follows (edited from Bolton (1987)):

**DPW** Dorsal Petiole Width. Maximum width of petiole in dorsal view.

**ED** Eye Diameter. Maximum diameter of eye with head positioned in profile view, such that anterior and posterior eye margins are in same plane of focus.

**HL** Head length. Length of head capsule, excluding mandibles, measured by a straight line from anterior clypeal margin to mid-point of a line drawn across posterior margin of head.

**HW** Head width. Maximum width of head, in full-face view, measured behind eyes (excluding eyes).

**ML** Mesosomal length. Maximum diagonal length of mesosoma in profile view, measured from posterodorsal border of pronotal flange to posterior basal angle of metapleuron.

**PW** Pronotal width. Maximum width of pronotum in dorsal view.

**SL** Scape length. Maximum length of antennal scape excluding basal constriction and condylar bulb.

**TL** Total length. Roughly measured from anterior margin of head to tip of gaster in stretched specimens.

**CI** Cephalic index. HW/HL × 100.

**DPI** Dorsal petiole index. DPW/PL × 100.

**OI** Ocular Index. ED/HW × 100.

**SI** Scape index. SL/HW × 100.

Abbreviations of the type depositories are as follows:

**MHNG**Muséum d’histoire naturelle, Geneva, Switzerland.

**SKYC** Seiki Yamane’s Collection at Kitakyushu Museum of Natural History and Human History, Japan.

**THNHM** Natural History Museum of the National Science Museum, Thailand.

### Results

#### Recurvidris lekakuli sp. n.

Taxon classificationAnimaliaHymenopteraFormicidae

http://zoobank.org/80A6A0F0-9D84-4B4C-865C-6831C7C7239B

Figures 1–5

##### Type.

**Holotype**: worker (THNHM-I-01219, THNHM), West Thailand, Kanchanaburi Province, Thong Phaphum District, Ban Sahakhon Nikhom, dry evergreen forest (DEF), 14.76255N, 98.80966E, 13.VII.2018, W. Jaitrong leg., WJT130718-07. **Paratypes**: three workers (THNHM-I-01220 to THNHM-I-01222, THNHM), same data as holotype; one worker (THNHM-I-01249, THNHM), West Thailand, Kanchanaburi Province, Thong Phaphum District, Ban Sahakhon Nikhom, dry evergreen forest (DEF), 14.76255N, 98.80966E, 26.VIII.2018, C. Sathiandee leg.; five workers (THNHM-I-02617, MHNG, SKYC, THNHM), West Thailand, Kanchanaburi Province, Thong Phaphum District, Ban Sahakhon Nikhom, dry evergreen forest (DEF), 14.76255N, 98.80966E, 6.XI.2018, W. Jaitrong leg., WJT061118-12; one worker (THNHM-I-02618, THNHM), West Thailand, Kanchanaburi Province, Thong Phaphum District, Ban Sahakhon Nikhom, dry evergreen forest (DEF), 14.76255N, 98.80966E, 6.XI.2018, C. Sathiandee leg., WJT1/5.

**Figures 1–5.**
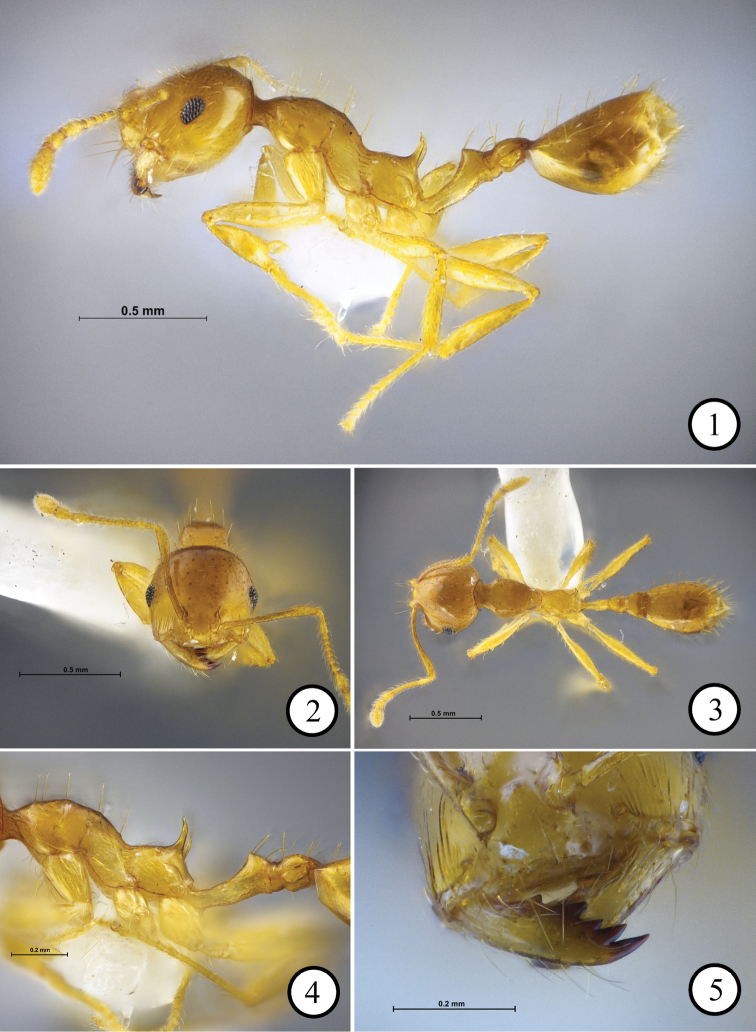
*Recurvidrislekakuli* sp. n., holotype worker (THNHM-I-01219) **1** body in profile **2** head in full-face view **3** body in dorsal view **4** mesosoma, petiole and postpetiole in profile **5** right mandible showing mandibular dentition.

##### Measurements and indices.

Holotype and four paratype workers (n = 5): DPW 0.10–0.12, ED 0.12–0.13, HL 0.50–0.56, HW 0.50–0.56, PW 0.23–0.26, ML 0.69–0.73, SL 0.53–0.56, TL 2.28–2.34, CI 97–100, DPI 30–35, OI 22–27, SI 100–107.

##### Diagnosis.

Head in full-face view round, almost as long as broad; masticatory margin of mandible with four sharp teeth, fourth (basal) tooth almost as large as third tooth; basal margin with a small tooth; propodeal declivity lacking infradental lamella or ridge linking propodeal spine to metapleural lobe; head, promesonotum, propodeum, petiolar node, postpetiole and gaster entirely smooth and shiny; mesopleuron and peduncle of petiole superficially reticulate with slightly smooth and shiny interspaces; propodeal dorsum with a pair of very short appressed hairs in front of spiracles.

##### Description

(Holotype and paratypes). Head in full-face view round and almost as long as broad, with posterior margin convex. Eye with seven ommatidia along longest axis. Antennal scape extending posteriorly slightly beyond posterolateral corner of head. Masticatory margin of mandible with four sharp teeth, fourth (basal) tooth as large as third tooth; basal margin with a small tooth. Clypeus without paired carinae, its anterior margin almost straight. Promesonotum in profile strongly convex dorsally and sloping gradually to metanotal groove. Propodeum in profile with almost straight dorsal outline; propodeal spines very slender, divergent, and in posterior view very narrow. Propodeal declivity lacking infradental lamella or ridge linking propodeal spine to metapleural lobe. Peduncle of petiole in profile relatively long, with its dorsal outline concave and ending posteriorly in right angle; its ventral outline convex with long acute subpetiolar process.

Head entirely smooth and shiny, lacking sculpture except some short longitudinal rugulae near mandiblular base. Antennal scape smooth and shiny. Promesonotum smooth and shiny; mesopleuron superficially reticulate with smooth and shiny interspaces; entire propodeum including propodeal spine smooth and shiny. Peduncle of petiole superficially reticulate with smooth and shiny interspaces; petiolar node entirely smooth and shiny; postpetiole entirely smooth and shiny; legs smooth and shiny. Gaster smooth and shiny.

Head with relatively dense short hairs; promesonotum with sparse longer hairs (8–10 hairs); longest pronotal hairs 0.13–0.15 mm long; propodeum dorsally with a pair of very short decumbent or appressed hairs (these hairs missing in two paratypes). Petiole with two dorsal pairs of long hairs. Postpetiole with two dorsal pairs of long hairs. Body colour yellow.

##### Etymology.

The specific name is dedicated to the late Dr. Boonsong Lekakul, who was the most excellent specialist in zoological sciences in Thailand and helped and inspired many young biologists.

##### Comparative notes.

*Recurvidrislekakuli* is closely related to *R.chanapaithooni* Jaitrong & Wiwatwitaya, 2015; *R.kemneri* Bolton, 1992; *R.nigrans* Zettel, 2008 and *R.proles* Bolton, 1992 in having the following characteristics: masticatory margin of mandible with a series of four sharp teeth (acute basal tooth), basal margin of mandible with a small tooth; propodeum without infradental lamella or ridge linking the propodeal spine to metapleural lobe; head smooth and shining. Among them *R.lekakuli* is more similar in general appearance to *R.chanapaithooni* and *R.kemneri* than to *R.nigrans* and *R.proles*, the former two sharing the clear yellow body that is unicolorous (black to dark brown in *R.nigrans* and *R.proles*). *Recurvidrislekakuli* is easily separated from *R.chanapaithooni* by the following characteristics: clearly larger body (TL 2.28–2.34 mm, HW 0.50–0.56 mm in *R.lekakuli*; TL 2.00–2.10 mm, HW 0.38–0.41 mm in *R.chanapaithooni*); mesopleuron largely smooth and shiny, only partly superficially reticulate (strongly reticulate in *R.chanapaithooni*); petiole relatively longer (DPI 30–35 in *R.lekakuli*; 42–43 in *R.chanapaithooni*); petiolar node clearly smooth and shiny (petiole entirely reticulate in *R.chanapaithooni*); propodeal dorsum with a pair of very short appressed hairs (with 2 pairs of standing hairs in *R.chanapaithooni*). *Recurvidrislekakuli* differs from *R.kemneri* by the clypeus clearly smooth and shiny (median portion of clypeus weakly bicarinate in *R.kemneri*) and having a pair of very short appressed hairs on propodeal dorsum (without hairs in *R.kemneri*). The new species is also similar to *R.glabriceps* Zhou, 2000 from China, but the latter lacks a small tooth on basal margin of mandible.

##### Bionomics.

**Figure 6.**
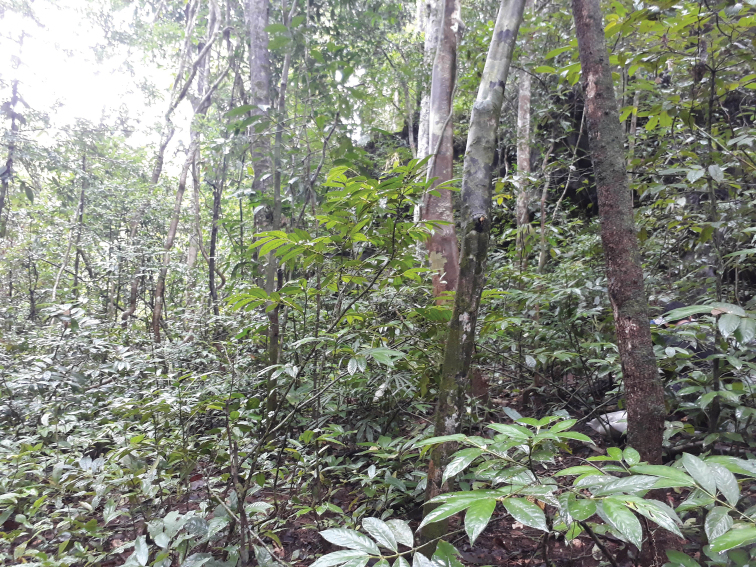
Type locality of *R.lekakuli* sp. n. at Ban Sahakhon Nikhom, Kanchanaburi Province, Thong Phaphum District, West Thailand, dry evergreen forest.

The type series was collected from leaf litter on the forest floor in a dry evergreen forest (Figure 6) near a stream.

##### Distribution.

*Recurvidrislekakuli* has been known only from the type locality. The most closely related species, *R.chanapaithooni* was recorded from eastern and southern Thailand (Jaitrong and Wiwatwitaya 2015). This species is very probably sympatric with *R.lekakuli* in at least the dry evergreen forest in western and/or southern Thailand.

### Key to Asian species of the genus *Recurvidris* based on worker caste

**Table:** 

1	Masticatory margin of mandible with 4 or 5 teeth, basal (fourth or fifth) tooth distinctly larger than preceding tooth, acute to bidenticulate; head behind level of frontal lobes sculptured (opaque, reticulate, reticulate-punctate); propodeal declivity with narrow infradental lamella or ridge linking propodeal spine to metapleural lobe	**2**
–	Masticatory margin of mandible with 4 teeth, basal (fourth) tooth at most only slightly larger than third; head behind level of frontal lobes unsculptured; propodeal declivity lacking infradental lamella or ridge linking propodeal spine to metapleural lobe	**7**
2	Masticatory margin of mandible with 5 teeth	**3**
–	Masticatory margin of mandible with 4 teeth	**4**
3	Dorsum of head finely and densely reticulate-punctate everywhere, dull and opaque; disc of pronotum finely and densely sculptured; subpetiolar process as a short tooth; small species (HW 0.39, see Bolton 1992)	***R.williami* Bolton, 1992**
–	Dorsum of head only with very fine superficially reticulate patterning, glossy; disc of pronotum glassy smooth; subpetiolar process as a long spine; large species (HW 0.45–0.56, see Bolton 1992; Jaitrong and Wiwatwitaya 2015)	***R.browni* Bolton, 1992**
4	Basal tooth of mandible acute apically	**5**
–	Basal tooth of mandible bidenticulate apically, may appear as abruptly truncated in worn mandible	**6**
5	Propodeal dorsum with only faint superficial sculpture and with a pair of short decumbent hairs	***R.pickburni* Bolton, 1992**
–	Propodeal dorsum with fine dense reticulate-rugulae and without a pair of short decumbent hairs	***R.nuwa* Xu & Zheng, 1995**
6	In profile view, propodeal spine and petiolar peduncle relatively short and stout (Figure 7); with head in full-face view, occipital corner narrowly rounded; postpetiole in dorsal view 1.6–1.8 times as broad as petiolar node (Bolton, 1992)	***R.recurvispinosa* Forel, 1890**
–	In profile view, propodeal spine and petiolar peduncle relatively long and narrow (Figure 8); with head in full-face view, occipital corner broadly rounded; postpetiole in dorsal view 1.3–1.4 times as broad as petiolar node (Bolton, 1992)	***R.hebe* Bolton, 1992**
7	Propodeal dorsum with 1–2 standing (erect) hairs	**8**
–	Propodeal dorsum with 1–2 pairs of very short decumbent or appressed hairs, without standing hairs.	**10**
8	Head and gaster dark brown, much darker than the yellowish mesosoma	***R.proles* Bolton, 1992**
–	Body color uniformly yellow	**9**
9	Basal margin of mandible unarmed; head relatively long (CI < 90)	***R.glabriceps* Zhou, 2000**
–	Basal margin of mandible armed with a small tooth which is widely separated from basal tooth; head relatively short and broad (CI ≥ 100)	***R.chanapaithooni* Jaitrong & Wiwatwitaya, 2015**
10	Body color uniformly blackish brown; in profile propodeal spine broad at base	***R.nigrans* Zettel, 2008**
–	Body yellowish brown; in profile propodeal spine narrow at base	**11**
11	Entire propodeum and dorsum of petiolar peduncle smooth and shiny; propodeal spine long, slightly longer than longest pronotal hairs	***R.lekakuli* sp. n.**
–	Dorsum of propodeum superficially sculptured; dorsum of petiolar peduncle finely reticulate; propodeal spine short, shorter than longest pronotal hairs	***R.kemneri* (Wheeler & Wheeler, 1954)**

**Figures 7–8.**
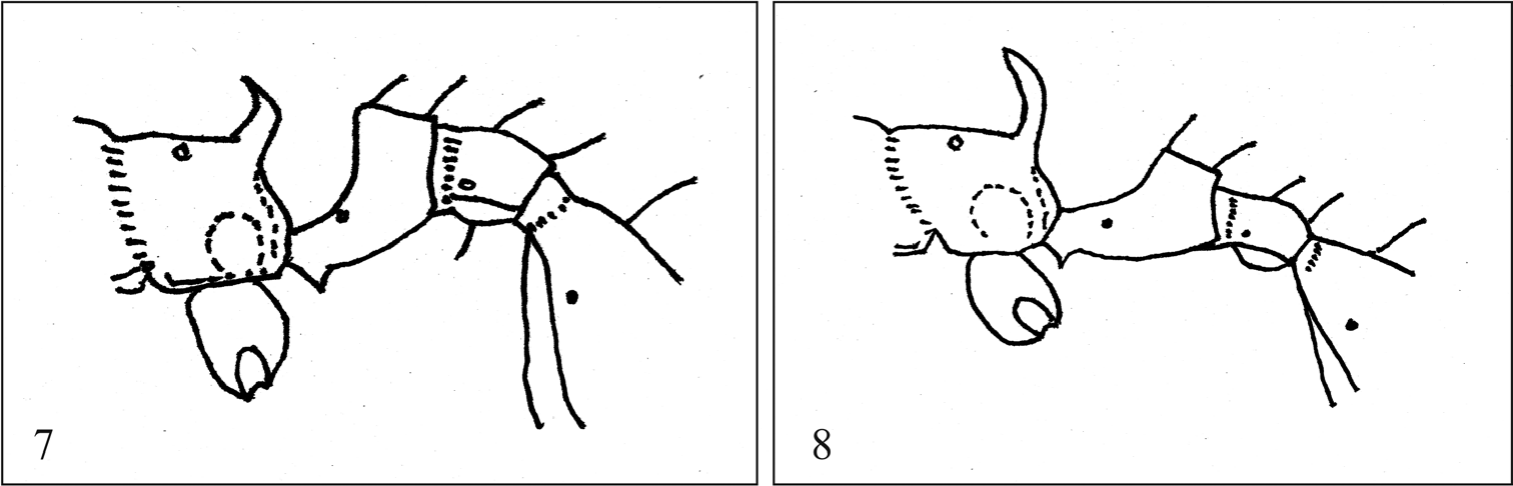
Propodeal spine and petiolar peduncle, edited from Bolton (1992)**7***R.recurvispinosa* (Forel, 1890) **8***R.hebe* Bolton, 1992.

## Supplementary Material

XML Treatment for Recurvidris lekakuli
